# Clinical Value of Prognostic Instruments to Identify Patients with an Increased Risk for Osteoporotic Fractures: Systematic Review

**DOI:** 10.1371/journal.pone.0019994

**Published:** 2011-05-18

**Authors:** Johann Steurer, Cyrill Haller, HansJörg Häuselmann, Florian Brunner, Lucas M. Bachmann

**Affiliations:** 1 Horten Center for Patient-Oriented Research and Knowledge Transfer, University of Zurich, Zurich, Switzerland; 2 Center for Rheumatology and Bone Disease Clinic, Zurich, Switzerland; 3 Department of Physical Medicine and Rheumatology, Balgrist University Hospital, Zurich, Switzerland; Marienhospital Herne - University of Bochum, Germany

## Abstract

**Background:**

With the broad availability of effective medications, identifying individuals bearing a higher risk for osteoporotic fractures has become an issue of major concern in modern medicine. In recent years various prognostic instruments have become available showing conflicting results regarding estimated risks for individual patients.

**Objective:**

To provide an overview of current evidence and of opportunities for further research.

**Methodology/Principal Findings:**

Systematic Review: We identified studies describing the development of instruments and all subsequent validations in electronic databases and reference lists of included studies. We screened for inclusion, read full papers and extracted data on salient clinical features, performance characteristics and quality in duplicate. Searches retrieved 5,275 records of which full texts of 167 papers were obtained after screening titles and abstract. We included 35 studies enrolling a total of 609,969 patients (median 2546) reporting on 31 derivations and 12 validations after assessing full texts. Median follow-up time was 4.1 years (IQR 3 to 7.7). Only four studies validated an instrument that was developed by another group. None of the existing instruments was validated more than once. The five most frequent included variables in the final model were age, body mass index, bone mass index, past history of falls, and maternal history of fractures. The methodological quality of the studies was moderate.

**Conclusion:**

There is a plethora of evidence available studying the association of risk profiles and the development of osteoporotic fractures. The small number of out-of-sample validations, the large variety of study characteristics, outcomes and follow-up periods impedes from deriving robust summaries and from conclusions regarding the clinical performance of many tools. First and foremost, future activity in this field should aim at reaching a consensus among clinical experts in respect to the existing instruments. Then we call for careful validations and expedient adaptations for local circumstances of the most promising candidates.

## Introduction

Osteoporotic fractures are a major cause of morbidity in the elderly and a considerable burden to health care services. Fractures necessitate hospitalizations in many cases and result in loss of function and autonomy of elderly people. Derived from present data it has been forecasted that 20% of 50-year old men and half of 50-year-old women will suffer from at least one clinical fracture during their remaining lifetime [1]. In Switzerland, the direct and indirect costs spent in caring for one patient with hip fracture is about 70.000 CHF (about 70.000 US $).

Preventive measures to reduce fractures therefore have high priority. Various lifestyle modifications and drugs have shown to reduce the decline in bone mineral density and the risk of osteoporotic fractures. An efficient strategy would be to identify persons with a moderate or high risk of fractures who might benefit from preventive or therapeutic measures. Measurement of bone mineral density alone may represent an inaccurate predictor of osteoporotic fractures and most fractures occur in women with normal bone mineral density [2]. A risk prediction rule to estimate the individual future fracture risk accurately would be helpful. In the last twenty years different risk prediction rules have been developed, but none of them, like other prediction rules, has found broad acceptance in medical practice so far.

The purpose of this review was to provide an overview of current evidence and of opportunities for further research. Moreover we explored whether setting-up a study developing a new prognostic instrument for the Swiss population is justified in light of the existing literature.

## Methods

### Literature search

In a first phase we searched for derivation studies in the following databases; Ovid MEDLINE (Ovid version, from inception to May 2009), and EMBASE and Scopus (from inception to May 2009). We used the following search terms: osteoporosis, bone loss, fractures, risk, model, logistic regression, decision, and validation. The detailed search strategy in Ovid Medline is available on **Table S5**. The search was conducted without restrictions to language or year of publication. We also hand-searched the bibliography of all studies ordered in full text.

In a second phase, after identifying the original derivation studies of prediction rules, we used these references to search in “ISI Web of Knowledge” (http://apps.isiknowledge.com) for corresponding validation studies. This database provides detailed information on how often and by whom a published paper has been cited (last access date on May 25, 2009). We assumed that all validations of an existing prediction rule would cite the derivation study.

### Selection criteria

We included studies describing the development or validation of prognostic instruments to identify individuals with an increased risk for osteoporotic fractures. The minimum requirement was that the study reported the final model, i.e. the selected indicators along with the strength of association with one of the following outcomes: hip/femur fracture, vertebral fracture or any/non-vertebral fracture. Moreover, a study had to provide a description about recruitment of participants and methods for variable selection.

### Study selection

#### Derivation studies

Two reviewers independently screened the titles and the abstracts of all retrieved references to identify derivation studies of prognostic instruments. Full text versions were ordered for all publications classified by any one of the reviewers as potentially relevant.

#### Validation studies

Two reviewers independently screened the titles and abstracts of all references to identify validation studies of one of the derivation studies. Full text versions were ordered for all potentially relevant publications.

### Data extraction

We developed article review forms that were pilot tested and revised before use. The two reviewers independently assessed each selected study by recording details about study sites, inclusion and exclusion criteria, number of eligible patients and number of included patients. In addition, the reviewers recorded the definitions for various risk factors and outcomes, kind of data collection (prospective or retrospective) and the statistical method to derive the prediction rule. We contacted one author of for further information about missing data but did not get any response [3].

The two reviewers determined the agreement about data extraction and final inclusion by consensus. If there was a disagreement, a third author made the conclusive decision.

## Results

**Figure 1:**
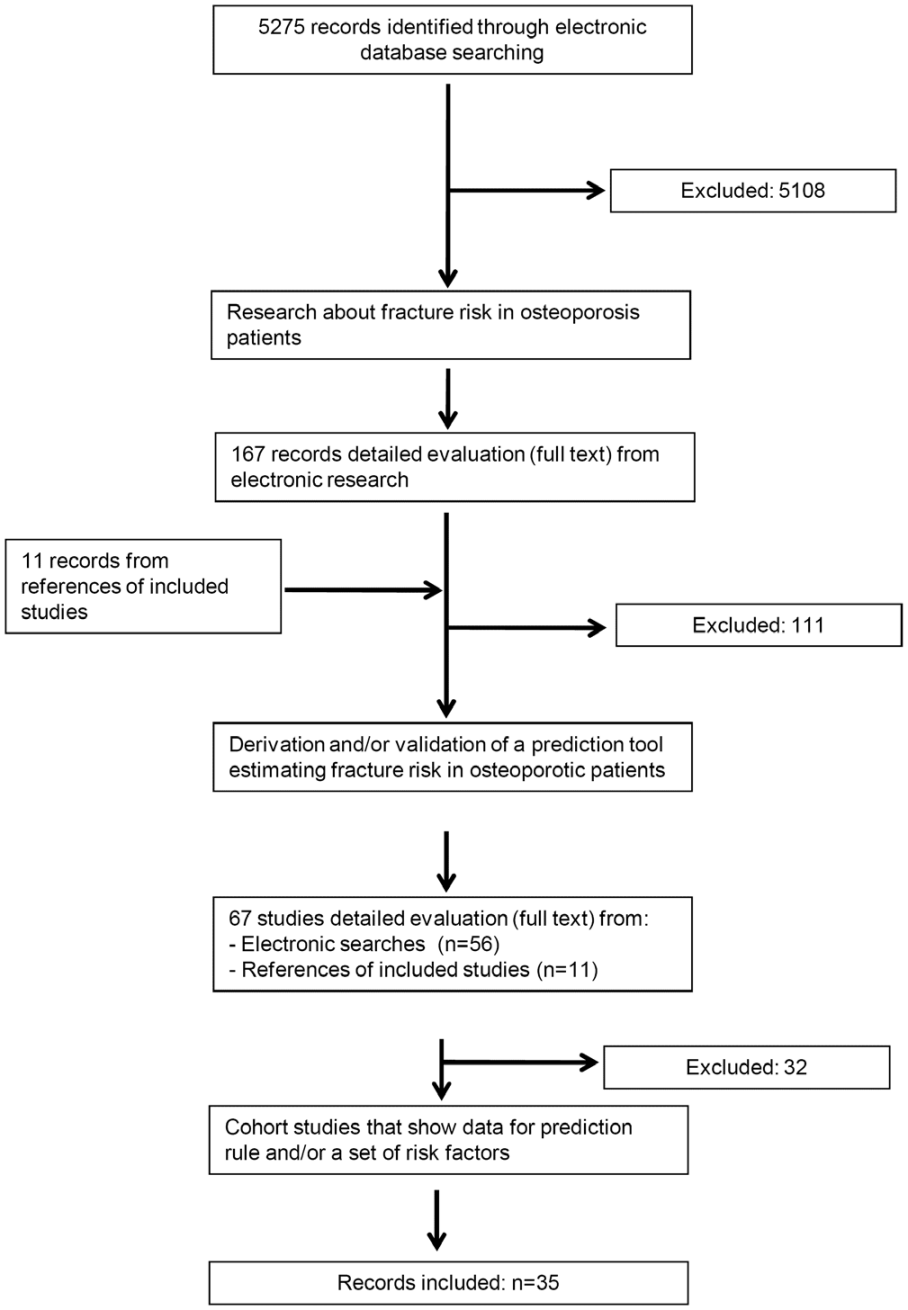
Flow of included studies.

In duplicate we screened 5275 records and ordered full texts of 167 papers. In total we included 35 studies reporting on 31 derivations [4], [5], [6], [7], [8], [9], [10], [11], [12], [13], [14], [15], [16], [17], [18], [19], [20], [21], [22], [23], [24], [25], [26], [27], [28], [29], [30], [31], [32], [33], [34] and 12 validations [8], [11], [13], [15], [23], [26], [32], [33], [35], [36], [37], [38] of instruments published between 1990 and 2009. In total 609,969 patients (median 2546, interquartile range: 1435 to 7654, range: 672 to 197,848) have been enrolled in the 35 studies. The study selection process is summarized in **Figure 1**. Median follow-up time was 4.1 years (interquartile range 3 to 7.7; range 1 to 15). In five studies duration of follow up was not reported [6], [13], [29], [30], [33]. Only four studies [35], [36], [37], [38] validated an instrument that was developed by another group. None of the existing instruments was validated more than once. We found substantial variation in respect to variable selection, definition of risk indicators, outcome assessment and forms of the final model. For details please refer to **Table S1**.

### Assessment of methodological quality

In 29 studies data were collected prospectively [4], [5], [7], [8], [9], [11], [12], [14], [15], [16], [17], [18], [19], [20], [21], [22], [23], [24], [25], [26], [27], [28], [29], [30], [31], [34], [35], [36], [37], six studies were based on registry data [6], [10], [13], [32], [33], [38]. Measurement of the outcome, vertebral fractures, was executed differently; in some studies the rate of vertebral fractures has been determined on clinical symptoms only [6], [8], [15], [16], [18], [23], [25], [31], [32], [33], [36], [37], [38], in others by radiography [4], [34], [35]. In six studies a random sample of participants were included [4], [5], [11], [14], [26], [35], in 3 participants were included in a consecutive manner [12], [18], [32] and in 17 studies an arbitrary sample has been evaluated [7], [8], [9], [15], [16], [19], [20], [21], [22], [23], [24], [25], [27], [28], [31], [34], [38] and in 9 no information about inclusion has been reported [6], [10], [13], [15], [17], [29], [30], [33], [37]. Variables were defined clearly and clinically sensible in 34 studies [4], [5], [6], [7], [8], [9], [10], [11], [12], [14], [15], [16], [17], [18], [19], [20], [21], [22], [23], [24], [25], [26], [27], [28], [29], [30], [31], [32], [33], [34], [35], [36], [37], [38]. In general the quality of the studies was moderate. The details are shown in **Table S2**.

### Assessed Outcomes

Hip fracture only was the assessed outcome in 12 studies [7], [8], [11], [16], [19], [21], [22], [26], [30], [34], [35], [36], non-vertebral fractures only in 3 studies [12], [24], [27] and all fractures in 19 studies [4], [5], [6], [9], [10], [14], [18], [20], [23], [28], [29], [31], [33], [37], [38]. The details are given in **Table S3**.

### Statistical methods applied in derivation studies

Logistic regression analysis was the most commonly used statistical model (n = 14) to derive the instrument [4], [5], [6], [7], [8], [10], [17], [19], [23], [25], [27], [28], [30], [31] and 12 studies used survival models (proportional hazard regression) [11], [12], [15], [18], [20], [21], [22], [24], [26], [32], [33], [34]. A Classification and Regression Tree (CART) algorithm was used in one study [9] and Poisson regression in another study [16].

### Variables in the final model

The five most frequent included variables in the final model were age, body mass index, bone mass index, past history of falls, and maternal history of fractures. Details on all included variables are given in **Table S4**.

## Discussion

### Main findings

Out of 35 studies enrolling more than 600,000 patients only four studies performed out of population validations of existing instruments. In many instances details of reporting of model development, form of the final model, descriptions of calibration, and discrimination and adjustments were unavailable. The five most frequent included variables in the final model were age, body mass index, bone mass index, past history of falls, and maternal history of fractures.

### Strength and Limitations

To our knowledge this is the first comprehensive assessment of prognostic instruments to identify patients with an increased risk for osteoporotic fractures. The strength of this study includes the application of robust systematic review methodology. We believe that our methods to search, identify and select relevant papers were sound. We made strenuous efforts to minimize the risk of selection bias. Relevant reports were searched systematically and without language restriction. The minimum requirement to be included in this review was that the study reported the final model, i.e. the selected indicators along with the strength of association with hip/femur fracture, vertebral fracture or any/non-vertebral fracture. The fact that we were unable performing a formal meta-analysis but were limited to systematic description alone is another limitation of this study. Study characteristics, outcomes and follow-up periods ranged substantially and impeded us thus from providing a statistically robust and clinically meaningful summary.

It may be argued that we did not include an eminent study, describing the development of the FRAX algorithm. This exclusion resulted after careful after a lengthy discourse among the authors. We finally refrained from including the FRAX algorithm in the study, because it failed to provide the minimum of information. We think that reports on prognostic models should always allow the reader to see the final form of the model and should allow re-calculating estimated risks for an individual patient. The provision of the model in a web interface is laudable but no substitute for complete reporting.

### Implications for Research

Two issues emerge from this review. The time has come for clinical experts to reach consensus about the validity of the existing evidence. We propose Delphi-type consensus studies that allow securing experts views on the correct type and form of input into these models. Once this has been assessed the available instruments should be checked against this consensus. If no instrument fulfilling the expert consensus exists, the international societies and other relevant healthcare bodies should set the research agenda for a well designed, carefully executed and validly analysed primary study. Researches should execute the studies carefully and provide an optimal reporting. Second, the models should be adapted to local circumstances, e.g. by careful calibration. Finally, impact studies should assess the transfer value of these instruments in practice.

### Conclusion

There is a plethora of evidence available studying the association of risk profiles and the development of osteoporotic fractures. The small number of out-of-sample validations, the large variety of study characteristics, outcomes and follow-up periods impedes from deriving robust summaries and from conclusions regarding the clinical performance of many tools. First and foremost, future activity in this field should aim at reaching a consensus among clinical experts in respect to the existing instruments. Then we call for careful validations and expedient adaptations for local circumstances of the most promising candidates.

## Supporting Information

Table S1 Descriptives of included Studies.
(DOC)

Table S2 (a) Methodological features of derivation studies. (b) Methodological features of validation studies.
(DOC)

Table S3 Outcomes.
(DOC)

Table S4 Variables included in the final model.
(DOC)

Table S5 Search history.
(DOC)
