# Correction: Clinical Value of Prognostic Instruments to Identify Patients with an Increased Risk for Osteoporotic Fractures: Systematic Review

**DOI:** 10.1371/annotation/7596ad0d-c602-4e5f-a7ae-caf7d5802720

**Published:** 2011-08-22

**Authors:** Johann Steurer, Cyrill Haller, HansJörg Häuselmann, Florian Brunner, Lucas M. Bachmann

The correct funding information is: “This work was sponsored by an unrestricted grant of the Osteoporosis Platform of the Swiss Society for Rheumatology. The funder had no role in study design, data collection and analysis, decision to publish, or preparation of the manuscript." 

