# Supplementary material for: Clinical Value of Prognostic Instruments to Identify Patients with an Increased Risk for Osteoporotic Fractures: Systematic Review
Source: PLoS One. 2011 May 18;6(5):e19994. doi: 10.1371/journal.pone.0019994 (PMC3097232; doi:10.1371/journal.pone.0019994)
Supplement: Table S1 — Descriptives of included Studies. (DOC) [file pone.0019994.s001.doc]

**Table 1** Descriptives of included Studies

| **Author/Year** | **Study site** | **Home-dwelling (H) Nursery-home (N)** | **No. of eligible participants** | **No. of included participants** | **Inclusion criteria** | **Exclusion criteria** | **Outcome** | **Follow-up** |
| --- | --- | --- | --- | --- | --- | --- | --- | --- |
|  |  |  |  |  |  |  |  |  |
| Ahmed L.A. 2006 | Tromso | NS | 27'159 | 1'410 | age 65 + attended | missing BMD values history of previous hip fracture | hip fracture | max. 5 years |
| Albrand G. 2003 | Lyon, France | H | 1'039 | 672 |  |  | all fractures excluding  major trauma fractures and fractures of toes and hands | 5.3 years average |
| Barrett-Connor E. 2005 | USA | NS | NS | 197'848 | age 50 + ≥ 6 months past the last menstrual period no diagnosis for osteoporosis no BDM within 12 months no bone-specific medications |  | self reported fracture of  hip, rib, spine, wrist, arm | 1 year |
| Bensen R. 2005 | Canada | NS | 3'426 | 3'373 |  | multiple fractures | vertebral fracture non-vertebral fracture hip fracture | NS |
| Black D.M. 2001 | USA | H | 9'704 |  | age 65 + | previous hip fracture bilateral hip replacement | hip fracture | 5 years |
| Burger H. 1999 | Netherlands | H/N | 10'275 | 5'208 | complete follow up |  | hip fracture | 3.8 years average |
| Chen Y.T. 2007 | mostly USA | H | 200'160 | 124'988 | 50 - 64 years old women | unreachable, deceased,  BMD measurement by AccuDEXA | all fracture | 3 years |
| Colón-Emeric C.S. 2002 | Duke, Iowa | NS | 7'654 | 7'654 | age 65 + | previous fracture | hip fracture any fracture | 10 years |
| Cummings S.R. 1995 | USA | NS | 9'516 | 9'516 | age 65 + able to walk white | bilateral hip replacement earlier hip fracture | hip fracture | 4.1 years average |
| Dargent-Molina P. 2002 | France | H | 7'575 | 6'933 |  | hip fracture bilateral hip replacement prolonged corticotherapy immobilization | hip fracture | ≥ 4 years |
| Diéz-Peréz A. 2007 | Spain | H | 5'201 | 5'146 | age 65 + informed consent |  | non-vertebral fracture | 3 years |
| Elffors L. 1993 | Europe | NS | 3.1 Mio. |  | age 50 + |  | hip fracture | 1 year |
| Ettinger B. 2005 | California | NS | > 400'000 | 14'528 | age 45 + fracture in hip, humerus or wrist |  | hip fracture humerus-, wrist fracture | NS |
| Fujiwara S. 2008 | Japan | NS | 2'596 | 2'596 |  |  | hip fracture distal radius fracture proximal humerus fracture clinical spinal fracture | 4 years |
| Girman C.J. 2002 | Maryland | N | 1'427 | 1'427 | age 65 + at least one wrist/forearm free of prostitic implants able to have BMD measurement | terminal cancer or bone metastases comatose significant open skin lesions admitted for rehab only | any fracture | 18 months |
| Guessous I. 2008 | Switzerland | NS | 7'609 | 6'174 | 70-85 y. old | history of hip fracture bilateral hip replacement renal failure active cancer or dementia | hip fracture non-vertebral fracture | 3 years |
| Hans D. 2008 | Switzerland, France | H | 12'958 | 12'958 | 70 - 100 years old all information available |  | hip fracture | 3.2 ± 0.9 years |
| Hippisley-Cox J. 2009 | England, Wales | H | 2’391’576 | 2’357’895 | 30-85 years old | previous recorded fracture( hip, distal radius, vertebral), temporary residents, pts. with interrupted periods of registration, not valid Townsend deprivation score | 1. Composite: hip, distal radius, vertebral fracture) 2. hip fracture alone | minimum one year, until first fracture |
| Krege J.H. 2006 | USA | NS | 2'127 | 2'127 | present osteoporosis |  |  | 23 months |
| Kung A.W.C. 2007 | China | H | 1'435 | 1'435 | age 45 +, Southern Chinese,  ≥ 1 year past menopause, no therapy for osteoporosis |  | all osteoporotic fractures | 5.0 ± 2.3 years |
| Leslie W.D. 2008 | Mantiboa, Canada | NS | 20'579 | 20'579 | age 47.5 + |  | hip, spine, forearm, prox.  Humerus | 10 years |
| McGrother C.W. 2002 | Melton Mowbray,  Leicestershire, UK | H/N | 1'864 | 1'289 | responder |  | hip fracture | 3 years and 5½ years |
| Nguyen N.D. 2005 | Dubbo, Australia | H | 2'095 w / 1'581 m | 960w / 689 m | age 60 + |  | hip fracture | 12 years |
| Nguyen N.D. 2007 | Dubbo, Australia | H | 2'095 w / 1'581 m | 1'028w / 740 m |  |  | hip fracture | 13 years |
| Nguyen N.D. 2008 | Dubbo, Australia | H | 2'095 w / 1'581 m | 1'358 w / 858 m | age 60 + |  | all osteoporotic fractures | 15 years |
| Nguyen T.V. 2001 | Dubbo, Australia | H | 2'095 w / 1'581 m | 1'105 w /  739 m | age 60 + humerus-, forearm- or wrist fracture or no fracture |  | humerus-, forearm- or  wrist fracture | 7 years |
| Nguyen T.V. 2004 | Dubbo, Australia | H | 2'095 w / 1'581 m | 1256 w |  | bone active medication | any fracture | 8.4 years average |
| Porthouse J. 2004 | UK | H | 35'000 | 4'292 | females aged 70 + returned baseline questionnaire at least 1 risk factor present | terminally ill no informed consent possible | non-vertebral fracture hip fracture wrist fracture | 2 years |
| Robbins J. 2007 | USA | NS | NS | 93'676 for derivation 68'132 for validation |  |  | hip fracture | 5 years |
| Roux C. 2007 | multi-national | NS | NS | 2546 |  |  | non-vertebral fracture | 3 years |
| Torgerson D.J. 1996 | Scotland | H | 2'300 | 1'857 | returned questionnaire famale, 45-49 years old | address change death life threatening illness | non-vertebral fracture | 2 years |
| Turner L.W. 1998 | USA | H | 20'277 | 2'325 | age 50 + |  | non-vertebral fracture | NS |
| Turner L.W. 1998 | USA | NS | 17'464 | 953 | age 50 + |  | hip fracture | NS |
| Van Hemert A.M. 1990 | Netherlands | Ns | 1'167 | 855 | 45-64 years old | non-participants death | all fractures | 9 years |
| Van Staa T.P. 2006 | UK | H | 366'104 50'000 for validation | NS* | THIN database | oral glucocorticoids both THIN and GPRD | Femur/hip fracture vertebral fracture other osteoporotic fracture | NS |

*NS = not stated
