# Supplementary material for: Clinical Value of Prognostic Instruments to Identify Patients with an Increased Risk for Osteoporotic Fractures: Systematic Review
Source: PLoS One. 2011 May 18;6(5):e19994. doi: 10.1371/journal.pone.0019994 (PMC3097232; doi:10.1371/journal.pone.0019994)
Supplement: Table S2 — (a) Methodological features of derivation studies. (b) Methodological features of validation studies. (DOC) [file pone.0019994.s002.doc]

Table 2 a. Methodological features of derivation studies

Derivation studies

|  | **Definition of outcome clinical (C) radiological (R)** | **Definition of outcome vertebral (V) non-vertebral (N) hip fracture (H) others (specify)** | **Data collection prospective (P) retrospective (RET) registry (REG) not mentioned (N)** | **Sample random (R) consecutive (C) arbitrary (A) not mentioned (N)** | **Clear, clinically  sensible and responsible definition of the variables** | **Statistical methods logistig regression (LR) recursive partitioning (RP) others (specify)** |
| --- | --- | --- | --- | --- | --- | --- |
| Albrand G. 2003 | R | V/N/H | P | R | yes | LR |
| Barrett-Connor E. 2005 | other | V/N/H | P | R | yes | LR/ categories -> bivariate  associations |
| Bensen R. 2005 | C | V/N/H | REG | N | yes | LR |
| Black D.M. 2001 | C/R | H | P | A | yes | LR |
| Burger H. 1999 | C | H | P | A | yes | LR |
| Chen Y.T. 2007 | other | V/N/H | P | A | yes | CART |
| Colón-Emeric C.S. 2002 | other | V/N/H | REG | N | yes | LR |
| Cummings S.R. 1995 | R | H | P | A | yes | proportional-hazards analysis |
| Dargent-Molina P. 2002 | other | H | P | R | yes | Cox regression |
| Diéz-Peréz A. 2007 | C/R | N | P | C | yes | Cox proportional hazard regression models |
| Ettinger B. 2005 | other | N/H | REG | N | no | NS* |
| Girman C.J. 2002 | C/R | V/N/H | P | R | yes | classification and regression tree (CART) methodology |
| Guessous I. 2008 | C | N/H | P | A | yes | univariate COX model |
| Hans D. 2008 | C | H | P | A | yes | poisson regression model |
| Hippisley-Cox J. 2009 | C | H (no definition of vertebral fracture) | REG | C | yes | Cox proportional hazard model |
| Krege J.H. 2006 | C/R | V | P | N | yes | LR |
| Kung A.W.C. 2007 | C | V/N/H | P | C | yes | Cox proportional hazard regression models |
| McGrother C.W. 2002 | C/R | H | P | A | yes | LR |
| Nguyen N.D. 2007 | C/R | H | P | A | yes | Cox proportional hazard regression models |
| Nguyen N.D. 2008 | C/R | V/N/H | P | A | yes | Cox proportional hazard regression models |
| Nguyen N.D. 2005 | C/R | H | P | A | yes | Cox proportional hazard regression models |
| Nguyen T.V. 2004 | C | V/N/H | P | A | yes | LR |
| Nguyen T.V. 2001 | C/R | N | P | A | yes | Cox proportional hazard regression models |
| Porthouse J. 2004 | C | N/H/wrist | P | A | yes | LR |
| Robbins J. 2007 | C/R | H | P | R | yes | Cox proportional hazard regression models |
| Roux C. 2007 | C/R | N | P | A | yes | LR |
| Torgerson D.J. 1996 | other | V/N/H | P | A | yes | LR |
| Turner L.W. 1998 | other | V/N/H | P | N | yes | NS* |
| Turner L.W. 1998 | other | H | P | N | yes | LR |
| Van Hemert A.M. 1990 | C | V/N/H | P | A | yes | LR |
| Van Staa T.P. 2006 | C | V/N/H | REG | N | yes | Cox proportional hazard regression models |

*NS = not stated

**Table 2 b**. methodological features of validation studies

| ***Validation studies*** |  |  |  |  |  |  |
| --- | --- | --- | --- | --- | --- | --- |
|  | **Definition of outcome clinical (C) radiological (R)** | **Definition of outcome vertebral (V) non-vertebral (N) hip fracture (H) others (specify)** | **Data collection prospective (P) retrospective (RET) registry (REG) not mentioned (N)** | **Sample random (R) consecutive (C) arbitrary (A) not mentioned (N)** | **Clear, clinically  sensible and responsible definition of the variables** | **Statistical methods logistiC regression (LR) recursive partitioning (RP) others (specify)** |
| Ahmed L.A. 2006 | R | H | P | R | yes | Cox proportional hazard regression models, Kaplan-Meier curves |
| Burger H. 1999 | C | H | P | A | yes | LR |
| Dargent-Molina P. 2002 | other | H | P | R | yes | Cox regression |
| Elffors L. 1993 | C | H | P |  | yes | NS* |
| Ettinger B. 2005 | other | N/H | REG | N | no | NS* |
| Fujiwara S. 2008 | C | V/N/H | P | N | yes | NS* |
| Guessous I. 2008 | C | N/H | P | N | yes | univariate COX model |
| Hippisley-Cox J. 2009 | C | H (no definition of vertebral fracture) | REG | C | yes | D statistics, R^2^ statistic, ROC |
| Leslie W.D. 2008 | C | V/N/H | REG | A | yes | Statistica (V. 6.1) (linear regression, Kaplan-Meier curves) |
| Nguyen T.V. 2004 | C | V/N/H | P | R | yes | LR |
| Robbins J. 2007 | C/R | H | P | R | yes | Cox proportional hazard regression models |
| Van Staa T.P. 2006 | C | V/N/H | REG | N | yes | Cox proportional hazard regression models |

*NS = not stated
