# Supplementary material for: Clinical Value of Prognostic Instruments to Identify Patients with an Increased Risk for Osteoporotic Fractures: Systematic Review
Source: PLoS One. 2011 May 18;6(5):e19994. doi: 10.1371/journal.pone.0019994 (PMC3097232; doi:10.1371/journal.pone.0019994)
Supplement: Table S3 — Outcomes. (DOC) [file pone.0019994.s003.doc]

Table 3 Outcomes

|  |  |  | **Outcomes** |  |  |
| --- | --- | --- | --- | --- | --- |
| **Study** | **Risk factors** | **Def of RF** | **Hip/Femur** | **Vertebral** | **Any/Non-vertebral** |
|  |  |  |  |  |  |
| Albrand G. 2003 | Personal history of fragility fracture after 45 years |  | 3.33 (1.42, 7.79) | 3.33 (1.42, 7.79) | 3.33 (1.42, 7.79) |
|  | BMD total hip | ≤ 0.736 g/cm² | 3.15 (1.75, 5.66) | 3.15 (1.75, 5.66) | 3.15 (1.75, 5.66) |
|  | Physical activity score | ≤ 14 | 2.08 (1.17, 3.69) | 2.08 (1.17, 3.69) | 2.08 (1.17, 3.69) |
|  | Left grip strenght | ≤ 0.60 bar | 2.05 (1.15, 3.64) | 2.05 (1.15, 3.64) | 2.05 (1.15, 3.64) |
|  | Age | ≥ 65 years | 1.90 (1.04, 3.47) | 1.90 (1.04, 3.47) | 1.90 (1.04, 3.47) |
|  | Maternal history of fragility fracture |  | 1.77 (1.01, 3.09) | 1.77 (1.01, 3.09) | 1.77 (1.01, 3.09) |
|  | Past falls |  | 1.76 (1.00, 3.09) | 1.76 (1.00, 3.09) | 1.76 (1.00, 3.09) |
| Barrett-Connor E. 2005 | Ethnicity | White |  |  | RR (95% CI): 1.00 |
|  |  | Black |  |  | RR (95% CI): 0.52 (0.38, 0.70) |
|  |  | Asian |  |  | RR (95% CI): 0.32 (0.15, 0.66) |
|  |  | Hispanic |  |  | RR (95% CI): 0.95 (0.76, 1.20) |
|  |  | Native American |  |  | RR (95% CI): 0.87 (0.57, 1.32) |
|  | BMD | per 1 SD decrease in T score |  |  | RR (95% CI): 1.54 (1.48, 1.61) |
| Bensen R. 2005 | weight | < 57 kg (reference level: ≥ 57 kg) | 1.70 (0.719, 4.037) | 1.57 (1.035, 2.373) |  |
|  | Use of arms to rise | (RL: rise without arms) | 3.58 (1.173, 10.927) | 1.72 (0.981, 3.023) |  |
|  | Smoking status | (RL: no smoking) | 1.48 (0.485, 4.536) | 1.95 (1.199, 3.184) |  |
|  | Age | 65 - 69 y (RL: < 65 y) | 1.53 (0.505, 4.667) | 1.36 (0.805, 2.309) |  |
|  |  | 70 - 74 y (RL: < 65 y) | 0.98 (0.280, 3.421) | 1.28 (0.753, 2.174) |  |
|  |  | 75 - 79 y (RL: < 65 y) | 1.62 (0.451, 5.840) | 1.96 (1.096, 3.508) |  |
|  |  | 80 - 84 y (RL: < 65 y) | 1.52 (0.296, 7.780) | 0.93 (0.368, 2.334) |  |
|  |  | 85 + y (RL: < 65 y) | 4.36 (0.823, 23.078) | 1.32 (0.375, 4.631) |  |
|  | Previous fracture after 50 y | (RL: no fracture) | 1.08 (0.477, 2.463) | 1.37 (0.931, 2.012) |  |
|  | BMD | < -2.5 SD (RL: > -2.5 SD) | 0.17 (0.233, 12.253) | 1.85 (0.448, 7.676) |  |
|  | Maternal history of fracture | (RL: no fracture) |  | 0.85 (0.296, 2.412) |  |
| Black D.M. 2001 | Age | per 5 years after 65: |  |  |  |
|  |  | Assessment without BMD   evaluation | 1.6 (1.4, 1.8) Point score: 0-5 |  |  |
|  |  | Assessment with BMD   evaluation | 1.4 (1.3, 1.6) Point score: 0-5 |  |  |
|  | Fracture after age 50 | Assessment without BMD   evaluation | 1.7 (1.3, 2.3) Point score: 1 |  |  |
|  |  | Assessment with BMD   evaluation | 1.5 (1.1, 2.0) Point score: 1 |  |  |
|  | Maternal hip fracture after  age 50 | Assessment without BMD   evaluation | 1.5 (1.0, 2.3) Point score: 1 |  |  |
|  |  | Assessment with BMD   evaluation | 1.4 (0.9, 2.1) Point score: 1 |  |  |
|  | Weight | ≤ 125 lb (57 kg): |  |  |  |
|  |  | Assessment without BMD   evaluation | 1.8 (1.4, 2.5) Point score: 1 |  |  |
|  |  | Assessment with BMD   evaluation | 1.2 (0.9, 1.7) Point score: 1 |  |  |
|  | Current smoker | Assessment without BMD   evaluation | 1.5 (0.9, 2.5) Point score: 1 |  |  |
|  |  | Assessment with BMD   evaluation | 1.3 (0.8, 2.2) Point score: 1 |  |  |
|  | Chair test | uses arms to rise from chair: |  |  |  |
|  |  | Assessment without BMD   evaluation | 2.5 (1.6, 3.8) Point score: 2 |  |  |
|  |  | Assessment with BMD   evaluation | 2.3 (1.5, 3.5) Point score: 2 |  |  |
|  | Total hip T-score | ≥ -1 | 1.0 Point score: 0 |  |  |
|  |  | -1 to -2 | 2.7 (1.5, 5.0) Point score: 2 |  |  |
|  |  | -2 to -2.5 | 4.7 (2.5, 8.7) Point score: 3 |  |  |
|  |  | < -2.5 | 7.0 (3.9, 12,8) Point score: 4 |  |  |
| Chen Y.T. 2007 | Classification rules: | CART Criteria |  |  |  |
|  | With BMD: | 1. History of fracture OR |  |  |  |
|  |  | 2. BMD T-score ≤ -1.1 OR |  |  |  |
|  |  | 3. Fair or poor self-reported  health status |  |  |  |
|  | Without BMD: | 1. History of fracture OR |  |  |  |
|  |  | 2. Fair or poor self-reported  health status OR |  |  |  |
|  |  | 3. Past or never use of  hormone therapy |  |  |  |
| Colón-Emeric C.S. 2002 | Age | > 75 years | Duke cohort: HR (95% CI): 2.05 (1.50, 2.81)  Iowa cohort: HR (95% CI): 2.33 (1.52, 1.66) |  |  |
|  | BMI | lowest quartile | Duke cohort: HR (95% CI): 1.34 (0.99, 1.82)  Iowa cohort: HR (95% CI): 2.41 (1.63, 3.56) |  | Duke cohort: HR (95% CI): 1.23 (1.09, 1.56)  Iowa cohort: HR (95% CI): 1.28 (0.99, 1.65) |
|  | female sex |  | Duke cohort: HR (95% CI): 2.31 (1.49, 3.56)  Iowa cohort: HR (95% CI): 1.80 (1.10, 3.00) |  | Duke cohort: HR (95% CI): 1.83 (1.51, 2.38)  Iowa cohort: HR (95% CI): 1.63 (1.23, 2.17) |
|  | white race |  | Duke cohort: HR (95% CI): 2.81 (2.03, 3.89)  Iowa cohort: HR (95% CI): n/a |  | Duke cohort: HR (95% CI): 2.20 (1.74, 2.51)  Iowa cohort: HR (95% CI): n/a |
|  | stroke history |  | Duke cohort: HR (95% CI): 1.89 (1.19, 2.99)  Iowa cohort: HR (95% CI): 0.84 (0.37, 1.93) |  |  |
|  | cognitive impairment | SPMSQ score ≤ 7 | Duke cohort: HR (95% CI): 1.89 (1.57, 3.21)  Iowa cohort: HR (95% CI): 1.61 (0.71, 3.70) |  |  |
|  | Rosow-Breslau impairment | difficulty doing heavy  work, walking up stairs,  or unable to walk 1/2 mile | Duke cohort: HR (95% CI): 1.77 (1.28, 2.43)  Iowa cohort: HR (95% CI): 1.65 (1.10, 2.49) |  | Duke cohort: HR (95% CI): 1.28 (1.01, 1.44)  Iowa cohort: HR (95% CI): 1.14 (0.87, 1.48) |
|  | ADL difficultiy | dependant in one or  more Katz scale items |  |  | Duke cohort: HR (95% CI): 1.54 (1.13, 1.92)  Iowa cohort: HR (95% CI): 1.35 (0.88, 2.09) |
|  | use of Anti-epileptic drugs |  |  |  | Duke cohort: HR (95% CI): 2.21 (1.27, 3.04)  Iowa cohort: HR (95% CI): n/a |
| Cummings S.R. 1995 | Base model |  |  |  |  |
|  | Age | per 5 years | RR (95% CI): 1.5 (1.3, 1.7) |  |  |
|  | History of maternal hip fracture | vs. none | RR (95% CI): 2.0 (1.4, 2.9) |  |  |
|  | Increase in weight since age 25 | per 20% | RR (95% CI): 0.6 (0.5, 0.7) |  |  |
|  | Height at age 25 | per 6 cm | RR (95% CI): 1.2 (1.1, 1.4) |  |  |
|  | Self-rated health | per 1-point decrease | RR (95% CI): 1.7 (1.3, 2.2) |  |  |
|  | Previous hyperthyroidism | vs. none | RR (95% CI): 1.8 (1.2, 2.6) |  |  |
|  | Current use of long-acting benzodiazepines | vs. no current use | RR (95% CI): 1.6 (1.1, 2.4) |  |  |
|  | Current use of anticonvulsive drugs | vs. no current use | RR (95% CI): 2.8 (1.2, 6.3) |  |  |
|  | Current caffeine intake | per 190 mg/day | RR (95% CI): 1.3 (1.0, 1.5) |  |  |
|  | Walking for exercise | vs. not walking for exercise | RR (95% CI): 0.7 (0.5, 0.9) |  |  |
|  | On feet ≤ 4 hr/day | vs. > 4 hr/day | RR (95% CI): 1.7 (1.2, 2.4) |  |  |
|  | Inability to rise from chair | vs. no inability | RR (95% CI): 2.1 (1.3, 3.2) |  |  |
|  | Lowest quartile for distant depth perception | vs. other three | RR (95% CI): 1.5 (1.1, 2.0) |  |  |
|  | Low-frequency contrast sensitivity | per 1 SD decrease | RR (95% CI): 1.2 (1.0, 1.5) |  |  |
|  | Resting pulse rate > 80 bpm | vs. ≤ 80 bpm | RR (95% CI): 1.8 (1.3, 2.5) |  |  |
|  |  |  |  |  |  |
|  | Add fractures and bone density |  |  |  |  |
|  | Age | per 5 years | RR (95% CI): 1.4 (1.2, 1.6) |  |  |
|  | History of maternal hip fracture | vs. none | RR (95% CI): 1.8 (1.2, 1.6) |  |  |
|  | Increase in weight since age 25 | per 20% | RR (95% CI): 0.8 (0.6, 0.9) |  |  |
|  | Height at age 25 | per 6 cm | RR (95% CI): 1.3 (1.1, 1.5) |  |  |
|  | Self-rated health | per 1-point decrease | RR (95% CI): 1.6 (1.2, 2.1) |  |  |
|  | Previous hyperthyroidism | vs. none | RR (95% CI): 1.7 (1.2, 2.5) |  |  |
|  | Current use of long-acting benzodiazepines | vs. no current use | RR (95% CI): 1.6 (1.1, 2.4) |  |  |
|  | Current use of anticonvulsive drugs | vs. no current use | RR (95% CI): 2.0 (0.8, 4.9) |  |  |
|  | Current caffeine intake | per 190 mg/day | RR (95% CI): 1.2 (1.0, 1.5) |  |  |
|  | Walking for exercise | vs. not walking for exercise | RR (95% CI): 0.7 (0.5, 1.0) |  |  |
|  | On feet ≤ 4 hr/day | vs. > 4 hr/day | RR (95% CI): 1.7 (1.2, 2.4) |  |  |
|  | Inability to rise from chair | vs. no inability | RR (95% CI): 1.7 (1.1, 2.7) |  |  |
|  | Lowest quartile for distant depth perception | vs. other three | RR (95% CI): 1.4 (1.0, 1.9) |  |  |
|  | Low-frequency contrast sensitivity | per 1 SD decrease | RR (95% CI): 1.2 (1.0, 1.5) |  |  |
|  | Resting pulse rate > 80 bpm | vs. ≤ 80 bpm | RR (95% CI): 1.7 (1.2, 2.4) |  |  |
|  | Any fracture since age of 50 | vs. none | RR (95% CI): 1.5 (1.1, 2.0) |  |  |
|  | Calcaneal bone density | per 1 SD increase | RR (95% CI): 1.6 (1.3, 1.9) |  |  |
| Dargent-Molina P. 2002 | Age | + 5 years | RR (95% CI): 1.4 (1.3, 1.5) Point score: 0-3 |  |  |
|  | History of falling |  | RR (95% CI): 1.4 (0.9, 2.0) Point score: 1 |  |  |
|  | Tandem walk | able after several trials | RR (95% CI): 1.0 (0.7, 1.4) Point score: 0 |  |  |
|  | (vs. able without trial) | unable | RR (95% CI): 2.0 (1.0, 3.8) Point score: 2 |  |  |
|  |  | not performed | RR (95% CI): 1.6 (1.2, 2.0) Point score: 2 |  |  |
|  | Gait speed | - 1 SD | RR (95% CI): 1.4 (1.3, 1.5) Point score: 0-3 |  |  |
|  | Visual acuity | 5-7/10e | RR (95% CI): 1.9 (1.3, 2.8) Point score: 2 |  |  |
|  | (vs. 7/10e) | 3-4/10e | RR (95% CI): 2.7 (1.7, 4.2) Point score: 3 |  |  |
|  |  | 0-2/10e | RR (95% CI): 2.1 (1.6, 2.8) Point score: 3 |  |  |
| Diéz-Peréz A. 2007 | Age | per 1 year |  |  | HR (95% CI): 1.03 (1.01, 1.05) |
|  | Fall in previous year | vs. none |  |  | HR (95% CI): 1.70 (1.35, 2.15) |
|  | Family history (1st degree) of fracture | vs. none |  |  | HR (95% CI): 1.53 (1.17, 1.99) |
|  | Personal history of low trauma fracture | vs. none |  |  | HR (95% CI): 1.73 (1.36, 2.21) |
|  | Calcium intake (dairy products) | < 250 mg/d |  |  | HR (95% CI): 1.92 (1.30, 2.86) |
|  | QUI | for each 1 SD decrease |  |  | HR (95% CI): 1.31 (1.15, 1.49) |
| Ettinger B. 2005 | Thinness | BMI < 21 | RR: 1.3 |  |  |
|  | Current smoker |  | RR: 1.5 |  |  |
|  | Family history of hip fracture | mother | RR: 1.3 |  |  |
|  |  | sister | RR: 1.6 |  |  |
|  | Prior non-spinal fracture at  any age ≥ 50 years |  | RR: 1.5 |  |  |
|  | No. of prior fractures | 1 |  | RR: 3.2 | RR: 1.6 |
|  |  | ≥ 2 |  | RR: 8.0 | RR: 2.0 |
|  | BMD | per SD |  | RR: 2.0 | RR: 1.5 |
| Girman C.J. 2002 | Age | 65-74 years |  |  | Fracture rate (%): 7.63 Point score: 0 |
|  |  | 75-84 years |  |  | Fracture rate (%): 11.56 Point score: 1 |
|  |  | 85-94 years |  |  | Fracture rate (%): 13.70 Point score: 2 |
|  |  | 95+ years |  |  | Fracture rate (%): 15.52 Point score: 3 |
|  | Weight (pounds) | < 170 |  |  | Fracture rate (%): 13.77 Point score: 1 |
|  |  | ≥ 170 |  |  | Fracture rate (%): 4.35 Point score: 0 |
|  | Height (inches) | ≤ 58 |  |  | Fracture rate (%): 15.92 Point score: 2 |
|  |  | 58 > height ≤ 63 |  |  | Fracture rate (%): 13.04 Point score: 1 |
|  |  | > 63 |  |  | Fracture rate (%): 11.16 Point score: 0 |
|  | Locomotion unit | Independant |  |  | Fracture rate (%): 17.34 Point score: 2 |
|  |  | Supervision |  |  | Fracture rate (%): 8.54 Point score: 1 |
|  |  | At least limited assistence  needed |  |  | Fracture rate (%): 10.40 Point score: 0 |
|  | Fell in last 180 days | No |  |  | Fracture rate (%): 11.47 Point score: 0 |
|  |  | Yes |  |  | Fracture rate (%): 15.53 Point score: 1 |
|  | Activities of daily living score | ≤ 4 |  |  | Fracture rate (%): 15.90 Point score: 1 |
|  |  | > 4 |  |  | Fracture rate (%): 9.43 Point score: 0 |
|  | Minimum Data Set:  cognition scale score | ≤ 3 |  |  | Fracture rate (%): 14.42 Point score: 1 |
|  |  | > 3 |  |  | Fracture rate (%): 11.98 Point score: 0 |
|  | Urinary incontinence | Usually continent or usually   incontinent |  |  | Fracture rate (%): 12.21 Point score: 0 |
|  |  | Occasionally incontinent |  |  | Fracture rate (%): 18.94 Point score: 1 |
| Guessous I. 2008 | Quantative US stiffness index | Lower than 60 | HR (95% CI): 4.51 (2.98, 6,85) Score pionts: 7.5 |  | HR (95% CI): 4.51 (2.98, 6,85) Score pionts: 7.5 |
|  |  | 60-78 | HR (95% CI): 2.77 (1.85, 4.15) Score points: 5 |  | HR (95% CI): 2.77 (1.85, 4.15) Score points: 5 |
|  | Age | 81-85 years | HR (95% CI): 1.87 (1.28, 2.72) Score points: 3 |  | HR (95% CI): 1.87 (1.28, 2.72) Score points: 3 |
|  |  | 75-80 years | HR (95% CI): 1.44 (1.13, 1.84) Score points: 2 |  | HR (95% CI): 1.44 (1.13, 1.84) Score points: 2 |
|  | Recent fall |  | HR (95% CI): 1.40 (1.11, 1.76) Score points: 1.5 |  | HR (95% CI): 1.40 (1.11, 1.76) Score points: 1.5 |
|  | Failed chair test |  | HR (95% CI): 1.27 (0.88, 1.83) Score points: 1 |  | HR (95% CI): 1.27 (0.88, 1.83) Score points: 1 |
|  | Fracture history |  | HR (95% CI): 1.23 (0.98, 1.55) Score points: 1 |  | HR (95% CI): 1.23 (0.98, 1.55) Score points: 1 |
| Hans D. 2008 | age | per 1 unit variation | HR (95% CI): 1.16 (1.13, 1.19) |  |  |
|  | early menopause | no vs. yes | HR (95% CI): 1.20 (0.89, 1.61) |  |  |
|  |  | surgical menopause | HR (95% CI): 1.00 (0.74, 1.36) |  |  |
|  | weight | per 1 unit variation | HR (95% CI): 0.99 (0.98, 1.00) |  |  |
|  | height | per 1 unit variation | HR (95% CI): 1.01 (0.99, 1.03) |  |  |
|  | BMI | per 1 unit variation | HR (95% CI): 0.96 (0.93, 0.98) |  |  |
|  | Smoking | current smoking: no vs. yes | HR (95% CI): 1.71 (1.08, 2.69) |  |  |
|  |  | ever smoked: no vs. yes | HR (95% CI): 1.14 (0.84, 1.55) |  |  |
|  | BMD | per 1 unit variation | HR (95% CI): 1.86 (1.64, 2.11) |  |  |
|  | Previous non-vertebral  fracture | no vs. yes | HR (95% CI): 1.50 (1.20, 1.88) |  |  |
|  | HRT | prior exposure to estrogens no vs. yes | HR (95% CI): 1.52 (1.06, 2.16) |  |  |
|  |  | current exposure to estrogens no vs. yes | HR (95% CI): 1.28 (0.71, 2.27) |  |  |
|  | maternal hip fracture | no vs. yes | HR (95% CI): 1.24 (0.85, 1.82) |  |  |
|  | chair test success | no vs. yes | HR (95% CI): 2.04 (1.54, 2.71) |  |  |
|  | Glucocorticoids | 3 months in the past | HR (95% CI): 1.24 (0.79, 1.96) |  |  |
|  |  | current | HR (95% CI): 1.62 (0.86, 3.05) |  |  |
|  | current thyroid hormone use | no vs. yes | HR (95% CI): 1.06 (0.61, 1.84) |  |  |
|  | fall history | no vs. yes | HR (95% CI): 1.36 (1.08, 1.73) |  |  |
|  | Diabetes | no vs. yes | HR (95% CI): 1.77 (1.22, 2.59) |  |  |
|  | higher education | no vs. yes | HR (95% CI): 2.62 (0.79, 1.53) |  |  |
|  | sedentary | no vs. yes | HR (95% CI): 1.35 (1.08, 1.70) |  |  |
|  | parous | no vs. yes | HR (95% CI): 1.33 (1.00, 1.77) |  |  |
| Hippisley-Cox J. 2009 | **men:** |  |  |  | ALL FRACTURES (hip, dist radius, vertebral) |
|  | Ex-smoker | no vs yes | HR 0.99 (0.89-1.10) |  | 0.98 (0.92-1.05) |
|  | Current smoker light | < 10 cigarettes/day | 1.21 (1.05-1.40) |  | 1.13 (1.03-1.23) |
|  | Current smoker moderate | 10 – 19 cigarettes/day | 1.47 (1.26-1.71) |  | 1.20 (1.12-1.29) |
|  | Current smoker heavy | ≥ 20 cigarettes/day | 1.70 (1.44-2.00) |  | 1.36 (1.25-1.47) |
|  | alcohol:trivial | < 1 unit/day | 0.78 (0.71-0.87) |  | 0.93 (0.86-0.99) |
|  | light | 1-2 units/day | 0.77 (0.70-0.86) |  | 0.95 (0.89-1.02) |
|  | moderate | 3-6 units/day | 0.81 (0.70-0.93) |  | 1.05 (0.95-1.16) |
|  | heavy | 7-9 units/day | 0.82 (0.56-1.20) |  | 1.10 (0.89-1.36) |
|  | very heavy | > 9 units/day | 1.70 (1.20-2.43) |  | 1.62 (1.34-1.96) |
|  | rheumatoid arthritis | no vs yes | 1.81 (1.31-2.50) |  | 1.48 (1.15-1.90) |
|  | cardiovascular disease | no vs yes | 1.24 (1.13-1.37) |  | 1.29 (1.20-1.39) |
|  | Type 2 diabetes | no vs yes | 1.38 (1.18-1.62) |  | 1.20 (1.07-1.35) |
|  | Asthma | no vs yes | 1.31 (1.12-1.53) |  | 1.28 (1.17-1.41) |
|  | Current tricyclic antidepressants | no vs yes | 1.67 (1.38-2.01) |  | 1.36 (1.18-1.56) |
|  | current corticosteroids | no vs yes | 1.22 (0.99-1.51) |  | 1.46 (1.27-1.68) |
|  | history of falls | no vs yes | 2.66 (2.03-3.49) |  | 2.23 (1.80-2.75) |
|  | liver disease | no vs yes | 2.96 (1.75-5.01) |  | 2.86 (2.04-4.01) |
|  | **WOMEN** |  |  |  |  |
|  | HR low dose unopposed equine oestrogen | no vs yes | 0.80 (0.66-0.97) |  | 0.90 (0.81-0.99) |
|  | low dose unopposed non-equine oestrogen | no vs yes | 0.87 (0.74-1.03) |  | 0.90 (0.82-0.98) |
|  | high dose unopposed equine oestrogen | no vs yes | 0.65 (0.40-1.06) |  | 0.75 (0.62-0.92) |
|  | high dose unopposed non-equine oestrogen | no vs yes | 0.80 (0.48-1.33) |  | 0.76 (0.61-0.96) |
|  | cyclical low dose equine | no vs yes | 0.93 (0.72-1.19) |  | 0.92 (0.83-1.02) |
|  | cyclical low dose non-equine | no vs yes | 0.80 (0.60-1.07) |  | 0.94 (0.84-1.04) |
|  | cyclical high dose equine | no vs yes | 1.00 (0.45-2.23) |  | 1.14 (0.84-1.56) |
|  | cyclical high dose non-equine | no vs yes | 0.91 (0.62-1.33) |  | 0.81 (0.70-0.94) |
|  | continuous low dose equine | no vs yes | 1.12 (0.80-1.56) |  | 1.24 (1.06-1.45) |
|  | continuous low dose non-equine | no vs yes | 0.84 (0.59-1.20) |  | 1.03 (0.89-1.19) |
|  | continuous high dose non-equine | no vs yes | 0.82 (0.64-1.06) |  | 0.88 (0.78-0.99) |
|  | tibolone | no vs yes | 0.62 (0.37-1.03) |  | 0.92 (0.74-1.14) |
|  | Ex-smoker | no vs yes | 1.04 (0.97-1.11) |  | 1.02 (0.98-1.06) |
|  | Current smoker light | < 10 cigarettes/day | 1.18 (1.05-1.32) |  | 1.04 (0.98-1.11) |
|  | Current smoker moderate | 10 – 19 cigarettes/day | 1.32 (1.22-1.43) |  | 1.11 (1.06-1.17) |
|  | Current smoker heavy | ≥ 20 cigarettes/day | 1.55 (1.39-1.73) |  | 1.21 (1.13-1.29) |
|  | alcohol:trivial | < 1 unit/day | 0.88 (0.82-0.94) |  | 1.00 (0.97-1.03) |
|  | light | 1-2 units/day | 0.87 (0.81-0.94) |  | 1.02 (0.97-1.07) |
|  | moderate | 3-6 units/day | 0.95 (0.77-1.18) |  | 1.03 (0.92-1.14) |
|  | heavy | 7-9 units/day | 1.41 (0.81-2.45) |  | 1.48 (1.06-2.06) |
|  | very heavy | > 9 units/day | 2.25 (1.35-3.73) |  | 2.01 (1.48-2.71) |
|  | parental history osteoporosis | no vs yes | 1.06 (0.71-1.58) |  | 1.78 (1.54-2.07) |
|  | rheumatoid arthritis | no vs yes | 1.78 (1.56-2.03) |  | 1.31 (1.18-1.44) |
|  | cardiovascular disease | no vs yes | 1.17 (1.09-1.24) |  | 1.17 (1.12-1.22) |
|  | Type 2 diabetes | no vs yes | 1.67 (1.51-1.83) |  | 1.25 (1.17-1.34) |
|  | Asthma | no vs yes | 1.32 (1.21-1.44) |  | 1.29 (1.22-1.36) |
|  | Current tricyclic antidepressants | no vs yes | 1.34 (1.24-1.45) |  | 1.31 (1.25-2.38) |
|  | current corticosteroids | no vs yes | 1.18 (1.05-1.31) |  | 1.17 (1.09-1.26) |
|  | history of falls | no vs yes | 2.03 (1.80-2.29) |  | 1.82 (1.66-1.99) |
|  | menopausal symptoms | no vs yes | 1.13 (0.99-1.28) |  | 1.15 (1.07-1.24) |
|  | liver disease | no vs yes | 1.65 (1.13-2.42) |  | 1.79 (1.41-2.27) |
|  | gastrointestinal malabsorption | no vs yes | 1.10 (0.85-1.42) |  | 1.23 (1.06-1.43) |
|  | other endocrine disorder | no vs yes | 1.19 (1.01-1.40) |  | 1.11 (1.00-1.25) |
|  |  |  |  |  |  |
|  |  |  |  |  |  |
| Krege J.H. 2006 | Previous vertebral  fracture |  |  |  | SQ score probability = 0.021 x 3 |
|  | Back pain |  |  |  | self reported propability = 0.021 x 4 |
|  | Height loss |  |  |  | measurement at beginning and end of study (3 x each -> average) probability = 0.021 x 3 |
| Kung A.W.C. 2007 | Age | per 10-year increase | RR (95% CI): 2.2 (1.7,2.8) | RR (95% CI): 2.2 (1.7,2.8) | RR (95% CI): 2.2 (1.7,2.8) |
|  | BMI | < 19 kg/m² | RR (95% CI): 1.8 (1.0, 3.2) | RR (95% CI): 1.8 (1.0, 3.2) | RR (95% CI): 1.8 (1.0, 3.2) |
|  | Dietary calcium intake | < 400 mg/d | RR (95% CI): 3.1 (1.9, 5.2) | RR (95% CI): 3.1 (1.9, 5.2) | RR (95% CI): 3.1 (1.9, 5.2) |
|  | Fracture history |  | RR (95% CI): 2.2 (1.4, 3.5) | RR (95% CI): 2.2 (1.4, 3.5) | RR (95% CI): 2.2 (1.4, 3.5) |
|  | 1 or more falls in 12 months |  | RR (95% CI): 4.0 (2.5, 6.2) | RR (95% CI): 4.0 (2.5, 6.2) | RR (95% CI): 4.0 (2.5, 6.2) |
|  | Housebound |  | RR (95% CI): 3.7 (1.9, 7.2) | RR (95% CI): 3.7 (1.9, 7.2) | RR (95% CI): 3.7 (1.9, 7.2) |
|  | Outdoor walking | < 30 min/d | RR (95% CI): 1.8 (1.1, 3.0) | RR (95% CI): 1.8 (1.1, 3.0) | RR (95% CI): 1.8 (1.1, 3.0) |
|  | Use of walking aids |  | RR (95% CI): 4.2 (2.7, 6.7) | RR (95% CI): 4.2 (2.7, 6.7) | RR (95% CI): 4.2 (2.7, 6.7) |
|  | L1 - L4 spine BMD | per SD reduction | RR (95% CI): 1.5 (1.2, 1.8) | RR (95% CI): 1.5 (1.2, 1.8) | RR (95% CI): 1.5 (1.2, 1.8) |
|  | Femoral neck BMD | per SD reduction | RR (95% CI): 2.0 (1.6, 2.5) | RR (95% CI): 2.0 (1.6, 2.5) | RR (95% CI): 2.0 (1.6, 2.5) |
|  | Trochanter BMD | per SD reduction | RR (95% CI): 1.7 (1.4, 2.1) | RR (95% CI): 1.7 (1.4, 2.1) | RR (95% CI): 1.7 (1.4, 2.1) |
|  | Total hip BMD | per SD reduction | RR (95% CI): 1.7 (1.4, 2.0) | RR (95% CI): 1.7 (1.4, 2.0) | RR (95% CI): 1.7 (1.4, 2.0) |
| McGrother C.W. 2002 | Age | per 5 years increase  (1) 3 years predictive model (2) 5½ years predicitve model | (2) 1.44 (1.10, 1.9) |  |  |
|  | Weight | per 1 SD decrease | (1) 1.8 (1.12, 2.9) (2) 1.6 (1.17, 3.2) |  |  |
|  | Trunk maneuver | Succeeded: OR 1.0 | (1) Used hands or failed: 4.8 (1.52, 15.0)  excluded from the test: 1.3 (0.33, 4.87) |  |  |
|  | short term steroid use |  | (1) 6.5 (1.6, 26,7) |  |  |
|  | Epilepsy |  | (1) 16.1 (2.0, 125,0) (2) 12.2 (1.9, 76,9) |  |  |
|  | Kyphosis |  | (1) 3.6 (1.45, 8.85) |  |  |
|  | Poor circulation |  | (1) 4.6 (1.75, 11.76) |  |  |
|  | Reported poor health |  | (2) 3.5 (1.08, 11.4) |  |  |
| Nguyen N.D. 2005 | Women |  |  |  |  |
|  | Age | + 5 years | HR (95% CI): 2.0 (1.7, 2.3) |  |  |
|  | Weight | - 10 kg | HR (95% CI): 2.6 (2.0, 3.2) |  |  |
|  | FNBMD | - 0.12 g/cm² | HR (95% CI): 3.6 (2.9, 4.5) |  |  |
|  | FNvBMD | - 0.03 g/cm² | HR (95% CI): 2.0 (1.8, 2.4) |  |  |
|  | Prior fracture | Yes | HR (95% CI): 2.5 (1.4, 4.3) |  |  |
|  | Falls in the past 12 months | Yes | HR (95% CI): 2.0 (1.3, 3.2) |  |  |
|  | Postural sway | + 60 cm² | HR (95% CI): 1.6 (1.4, 1.8) |  |  |
|  | Quadriceps strenght | - 10 kg | HR (95% CI): 2.6 (1.9, 3.6) |  |  |
|  | Men |  |  |  |  |
|  | Age | + 5 years | HR (95% CI): 2.6 (2.0, 3.5) |  |  |
|  | Weight | - 10 kg | HR (95% CI): 1.6 (1.2, 3.2) |  |  |
|  | FNBMD | - 0.12 g/cm² | HR (95% CI): 3.4 (2.5, 4.6) |  |  |
|  | FNvBMD | - 0.03 g/cm² | HR (95% CI): 2.7 (2.0, 3.7) |  |  |
|  | Prior fracture | Yes | HR (95% CI): 7.2 (3.1, 17.0) |  |  |
|  | Falls in the past 12 months | Yes | HR (95% CI): 2.0 (1.0, 4.2) |  |  |
|  | Postural sway | + 60 cm² | HR (95% CI): 2.1 (1.6, 2.6) |  |  |
|  | Quadriceps strenght | - 10 kg | HR (95% CI): 2.3 (1.7, 3.2) |  |  |
| Nguyen N.D. 2007 | Women |  |  |  |  |
|  | Age | + 5 years | HR (95% CI): 1.95 (1.70, 2.22) |  |  |
|  | Weight | - 10 kg | HR (95% CI): 2.12 (1.70, 2.64) |  |  |
|  | Height | - 5 cm | HR (95% CI): 1.58 (1.34, 1.88) |  |  |
|  | BMI | - 5 kg/m² | HR (95% CI): 2.09 (1.57, 2.80) |  |  |
|  | FNBMD | - 0.12 g/cm² | HR (95% CI): 2.62 (2.21, 3.11) |  |  |
|  | LSBMD | - 0.20 g/cm² | HR (95% CI): 2.37 (1.83, 3.07) |  |  |
|  | Postural sway | + 60 cm² | HR (95% CI): 1.63 (1.41, 1.88) |  |  |
|  | Quadriceps strenght | - 10 kg | HR (95% CI): 1.89 (1.51, 2.37) |  |  |
|  | Daily calcium intake | - 300mg/d | HR (95% CI): 0.92 (0.75, 1.13) |  |  |
|  | Home physical activity | - 30 METS | HR (95% CI): 0.95 (0.78, 1.16) |  |  |
|  | Menopausal age | - 2 yrs | HR (95% CI): 1.03 (0.98, 1.09) |  |  |
|  | Prior fracture | each fracture | HR (95% CI): 4.23 (2.96, 6.04) |  |  |
|  | Falls in the past 12 months | each fall | HR (95% CI): 1.40 (1.20, 1.62) |  |  |
|  | Smoking | Yes | HR (95% CI): 0.91 (0.91, 1.42) |  |  |
|  | Men |  |  |  |  |
|  | Age | + 5 years | HR (95% CI): 2.31 (1.76, 3.03) |  |  |
|  | Weight | - 10 kg | HR (95% CI): 1.43 (1.05, 1.95) |  |  |
|  | Height | - 5 cm | HR (95% CI): 1.48 (1.14, 1.92) |  |  |
|  | BMI | - 5 kg/m² | HR (95% CI): 1.37 (0.81, 2.31) |  |  |
|  | FNBMD | - 0.12 g/cm² | HR (95% CI): 2.61 (1.95, 3.50) |  |  |
|  | LSBMD | - 0.20 g/cm² | HR (95% CI): 1.61 (1.10, 2.34) |  |  |
|  | Postural sway | + 60 cm² | HR (95% CI): 1.78 (1.37, 2.31) |  |  |
|  | Quadriceps strenght | - 10 kg | HR (95% CI): 1.84 (1.45, 2.33) |  |  |
|  | Daily calcium intake | - 300mg/d | HR (95% CI): 1.47 (0.90, 2.41) |  |  |
|  | Home physical activity | - 30 METS | HR (95% CI): 1.02 (0.79, 1.32) |  |  |
|  | Prior fracture | each fracture | HR (95% CI): 2.89 (1.85, 4.50) |  |  |
|  | Falls in the past 12 months | each fall | HR (95% CI): 1.42 (0.93, 2.16) |  |  |
|  | Smoking | Yes | HR (95% CI): 0.65 (0.32, 1.31) |  |  |
| Nguyen N.D. 2008 | Women |  |  |  |  |
|  | Age | + 5 years | HR (95% CI): 1.43 (1.34, 1.53) |  |  |
|  | Weight | - 10 kg | HR (95% CI): 1.25 (1.15, 1.37) |  |  |
|  | Height | - 5 cm | HR (95% CI): 1.19 (1.10, 1.29) |  |  |
|  | BMI | - 5 kg/m² | HR (95% CI): 1.25 (1.11, 1.40) |  |  |
|  | FNBMD | - 0.12 g/cm² | HR (95% CI): 1.68 (1.55, 1.82) |  |  |
|  | LSBMD | - 0.20 g/cm² | HR (95% CI): 1.68 (1.49, 1.89) |  |  |
|  | Daily calcium intake | - 350 mg/d | HR (95% CI): 0.94 (0.85, 1.04) |  |  |
|  | Home physical activity | - 30 METs | HR (95% CI): 0.97 (0.88, 1.07) |  |  |
|  | Current/ex-smoking | Yes | HR (95% CI): 1.05 (0.85, 1.29) |  |  |
|  | Prior fracture | plus one fracture (until 3) | HR (95% CI): 2.06 (1.87, 2.26) |  |  |
|  | Falls in the past 12 months | plus one fall (until 3) | HR (95% CI): 1.23 (1.10, 1.38) |  |  |
|  | Current use of steroid | Yes | HR (95% CI): 1.34 (0.35, 2.20) |  |  |
|  | Rheumatoid arthritis | Yes | HR (95% CI): 1.49 (1.00, 2.19) |  |  |
|  | Maternal history of osteoporosis | Yes | HR (95% CI): 1.07 (0.83, 1.36) |  |  |
|  | Paternal history of osteoporosis | Yes | HR (95% CI): 0.97 (0.59, 1.59) |  |  |
|  | Men |  |  |  |  |
|  | Age | + 5 years | HR (95% CI): 1.67 (1.48, 1.88) |  |  |
|  | Weight | - 10 kg | HR (95% CI): 1.31 (1.14, 1.51) |  |  |
|  | Height | - 5 cm | HR (95% CI): 1.28 (1.13, 1.44) |  |  |
|  | BMI | - 5 kg/m² | HR (95% CI): 1.30 (1.03, 1.65) |  |  |
|  | FNBMD | - 0.12 g/cm² | HR (95% CI): 1.62 (1.43, 1.83) |  |  |
|  | LSBMD | - 0.20 g/cm² | HR (95% CI): 1.54 (1.30, 1.82) |  |  |
|  | Daily calcium intake | - 350 mg/d | HR (95% CI): 1.08 (0.90, 1.31) |  |  |
|  | Home physical activity | - 30 METs | HR (95% CI): 0.90 (0.82, 0.99) |  |  |
|  | Current/ex-smoking | Yes | HR (95% CI): 1.14 (0.82, 1.59) |  |  |
|  | Prior fracture | plus one fracture (until 3) | HR (95% CI): 2.92 (2.43, 3.52) |  |  |
|  | Falls in the past 12 months | plus one fall (until 3) | HR (95% CI): 1.38 (1.13, 1.69) |  |  |
|  | Current use of steroid | Yes | HR (95% CI): 0.92 (0.38, 2.23) |  |  |
|  | Rheumatoid arthritis | Yes | HR (95% CI): 1.23 (0.47, 3.45) |  |  |
|  | Maternal history of osteoporosis | Yes | HR (95% CI): 1.03 (0.65, 1.63) |  |  |
|  | Paternal history of osteoporosis | Yes | HR (95% CI): 0.26 (0.04, 1.84) |  |  |
|  | multivariate analysis: |  |  |  |  |
|  | Model 1: | age+baseline BMD+ prior fracture+fall |  |  |  |
|  | age | each SD older | HR (95% CI): 1.25 (1.17, 1.30) |  |  |
|  | BMD | each SD lower | HR (95% CI): 1.36 (1.26, 1.47) |  |  |
|  | Prior fracture |  | HR (95% CI): 1.84 (1.68, 2.03) |  |  |
|  | previous falls |  | HR (95% CI): 1.22 (1.11, 1.36) |  |  |
|  | Model 2: | age+baseline weight+ prior fracture+fall |  |  |  |
|  | age |  | HR (95% CI): 1.34 (1.26, 1.43) |  |  |
|  | body weight |  | HR (95% CI): 1.10 (1.02, 1.19) |  |  |
|  | Prior fracture |  | HR (95% CI): 2.05 (1.87, 2.25) |  |  |
|  | previous falls |  | HR (95% CI): 1.22 (1.10, 1.36) |  |  |
| Nguyen T.V. 2001 | Men |  |  |  |  |
|  | Humerus fracture |  |  |  |  |
|  | Femoral neck BMD | - 0.1 g/cm² | RR (95% CI): 2.27 (1.16, 4.48) |  |  |
|  | Height loss | - 1 cm | RR (95% CI): n/a |  |  |
|  | Forearm and wrist fracture |  |  |  |  |
|  | Femoral neck BMD | - 0.1 g/cm² | RR (95% CI): 1.52 (1.01, 2.29) |  |  |
|  | Dietary calcium intake | - 300 mg/d | RR (95% CI): 1.98 (1.00, 3.58) |  |  |
|  | Height loss | - 1 cm | RR (95% CI): 1.15 (1.03, 1.34) |  |  |
|  | Falls | each | RR (95% CI): n/a |  |  |
|  | Women |  |  |  |  |
|  | Humerus fracture |  |  |  |  |
|  | Femoral neck BMD | - 0.1 g/cm² | RR (95% CI): 2.44 (1.72, 3.48) |  |  |
|  | Height loss | - 1 cm | RR (95% CI): 1.12 (1.02, 1.23) |  |  |
|  | Forearm and wrist fracture |  |  |  |  |
|  | Femoral neck BMD | - 0.1 g/cm² | RR (95% CI): 1.52 (1.22, 1.90) |  |  |
|  | Dietary calcium intake | - 300 mg/d | RR (95% CI): n/a |  |  |
|  | Height loss | - 1 cm | RR (95% CI): 1.08 (1.02, 1.16) |  |  |
|  | Falls | each | RR (95% CI): 1.89 (1.36, 2.61) |  |  |
| Porthouse J. 2004 | Age | per year increase | 1.09 (1.04, 1.13) |  | 1.03 (1.01, 1.05) |
|  | Previous fracture |  | 2.31 (1.31, 4.08) |  | 2.67 (2.10, 3.40) |
|  | Fall in the last 12 months |  | 2.92 (1.70, 5.01) |  | 2.06 (1.63, 2.59) |
|  | Current smoking |  | 0.69 (0.21, 2.25) |  | 0.69 (0.41, 1.15) |
|  | Maternal hip fracture |  | 1.68 (0.85, 3.31) |  | 0.86 (0.59, 1.25) |
|  | Poor/fair self-reported health |  | 1.40 (0.82, 2.40) |  | 1.04 (0.81, 1.34) |
|  | Low body weight (< 58kg) |  | 2.20 (1.28, 3.77) |  | 1.06 (0.82, 1.36) |
|  |  |  |  |  |  |
| Robbins J. 2007 | Age | per each year | 1.13 (1.11, 1.15) Point score: ½ per year > 50 |  |  |
|  | Self-reported health | Fair or poor vs. excellent | 2.38 (1.66, 3.40) Point score: 3 |  |  |
|  |  | Good vs. excellent | 1.22 (0.90, 1.66) Point score: 1 |  |  |
|  |  | Very good vs. excellent | 1.11 (0.83, 1.49) Point score: 0 |  |  |
|  | Height | per each inch | 1.11 (1.07, 1.16) Point score: ½ per inch > 64 |  |  |
|  | Weight | per each pound | 0.99 (0.98, 0.99) Point score: 1 per 25 lb >200 |  |  |
|  | Fracture on or after age 55 y | Not applicable vs. No | 1.01 (0.51, 2.02) Point score: 0 |  |  |
|  |  | Yes vs. No | 1.72 (1.41, 2.10) Point score: 2 |  |  |
|  | Race |  | Point score: 3 for 'White' |  |  |
|  |  | Unknown vs. White | 1.00 (0.47, 2.14) |  |  |
|  |  | Asian/Pacific Islander vs. White | 0.26 (0.10, 0.70) |  |  |
|  |  | American Indian vs. White | 1.60 (0.50, 5.10) |  |  |
|  |  | Hispanic vs. White | 0.32 (0.12, 0.86) |  |  |
|  |  | Black vs. White | 0.41 (0.24, 0.70) |  |  |
|  | Physical activity, MET's |  | Point score: 1 |  |  |
|  |  | 5-12 vs. ≤ 12 | 1.32 (1.04, 1.67) |  |  |
|  |  | <5 vs. ≤ 12 | 1.26 (0.97, 1.64) |  |  |
|  |  | Inactive 0 vs. ≤ 12 | 1.64 (1.24, 2.17) |  |  |
|  | Smoking status | current vs. never | 2.33 (1.71, 3.18) Point score: 3 |  |  |
|  |  | past vs. never | 0.96 (0.79, 1.17) Point score: 0 |  |  |
|  | Parent broke hip | Yes vs. No | 1.50 (1.20, 1.87) Point score: 1 |  |  |
|  | Corticosteroid use | Yes vs. No | 1.94 (1.16, 3.25) Point score: 3 |  |  |
|  | Use of hypoglycemic agent | Yes vs. No | 1.74 (1.17, 2.60) Point score: 2 |  |  |
| Roux C. 2007 | Intercept |  |  |  | -9.1119 |
|  | Age | years |  |  | OR: 1.038 |
|  | Height | cm |  |  | OR: 1.024 |
|  | femoral neck T-score |  |  |  | OR: 1.290 |
|  | Vitamin D | ng/ml |  |  | OR: 1.011 |
|  | prevalent vertebral fracture | number |  |  | OR: 1.113 |
|  | prevalen non-vert. fracture | yes: -1 no: 1 |  |  |  |
| Torgerson D.J. 1996 | Intercept |  |  |  | 98.69 |
|  | Previous fracture history | once |  |  | 2.10 (1.03, 4.26) |
|  |  | twice or more |  |  | 4.04 (1.72, 9.50) |
|  | BMD of spine | lowest quarter |  |  | 4.55 (1.53, 13.50) |
|  |  | 2nd quarter |  |  | 2.65 (0.83, 8.43) |
|  |  | 3rd quarter |  |  | 1.78 (0.51, 6.15) |
|  | History of hip fracture by  maternal grandmother |  |  |  | 3.70 (1.55, 8.85) |
|  | Not menstruating |  |  |  | 1.98 (1.02, 3.56) |
| Turner L.W. 1998 | Intercept |  | -5.4121 |  |  |
|  | Black |  | 0.058 (0.014, 0.242) |  |  |
|  | Hispanic |  | 0.432 (0.189, 0.998) |  |  |
|  | Age | ≥ 65 years | 7.451 (2.948, 18.834) |  |  |
|  | BMI | low (< 20) | 1.953 (1.160, 3.289) |  |  |
| Turner L.W. 1998 | Age | 65+ years old |  |  | 5.390 (3.273, 8.878) |
|  | BMI | < 20 |  |  | 1.427 (1.000, 2.037) |
|  | Black |  |  |  | 0.186 (0.100, 0.349) |
|  | Hispanic |  |  |  | 0.477 (0.295, 0.772) |
|  | physical activity | equivalent to 20 min. excercise: fewer than 2 times per week |  |  | 1.838 (1.293, 2.613) |
| Van Hemert A.M. 1990 | Age/Lactation | Age > 55 y and lactation > 12 mo |  | Coefficient(standart error):  1.3 (0.33) | Co(SE): 1.1 (0.27) |
|  |  | Age > 55 y and no children |  | Co(SE): 1.1 (0.45) | Co(SE): 1.1 (0.37) |
|  | Metacarpal bone density | > 81 % |  |  | Co(SE): -0.9 (0.27) |
|  |  | < 76% |  |  | Co(SE): -0.5 (0.23) |
|  | Height | < 160 cm |  | Co(SE): -1.1 (0.35) | Co(SE): -0.7 (0.27) |
|  | Diameter of forearm | < 48 mm |  |  | Co(SE): -0.7 (0.27) |
|  |  | > 50 mm |  |  | Co(SE): -0.6 (0.24) |
|  | Age/Metacarpal bone density | Age > 55 y and density < 47 mm² |  | Co(SE): 0.6 (0.34) |  |
|  | Age at menarche | ≥ 16 years |  | Co(SE): 0.6 (0.34) |  |
| Van Staa T.P. 2006 | Age | reference: 50-59 years | 60-69: RR (95% CI): 3.54 (2.98, 4.15) 70-79: RR (95% CI): 12.57 (10.81, 14.63) 80-89: RR (95% CI): 40.96 (35.33, 47.50) 90+: RR (95% CI): 71.27 (61.03, 83.24) | 60-69: RR (95% CI): 2.26 (1.85, 2.77) 70-79: RR (95% CI): 5.46 (4.54, 6.56) 80-89: RR (95% CI): 9.15 (7.60, 11.01) 90+: RR (95% CI): 7.95 (6.16, 10.26) | 60-69: RR (95% CI): 1.67 (1.59, 1.76) 70-79: RR (95% CI): 2.45 (2.33, 2.58) 80-89: RR (95% CI): 3.51 (3.33, 3.70) 90+: RR (95% CI): 3.83 (3.53, 4.15) |
|  | history of early menopause |  | RR (95% CI): 2.23 (1.12, 4.47) | RR (95% CI): 1.89 (0.47, 7.58) | RR (95% CI): 0.68 (0.33, 1.43) |
|  | BMI |  | <20: RR (95% CI): 1.91 (1.74, 2.09) ≥ 26: RR (95% CI): 0.63 (0.58, 0.69) | <20: RR (95% CI): 1.41 (1.16, 1.72) ≥ 26: RR (95% CI): 0.84 (0.74, 0.96) | <20: RR (95% CI): 1.21 (1.13, 1.31) ≥ 26: RR (95% CI): 0.84 (0.80, 0.88) |
|  | Smoking |  | RR (95% CI): 1.44 (1.33, 1.57) | RR (95% CI): 1.45 (1.26, 1.68) | RR (95% CI): 1.13 (1.08, 1.19) |
|  | Previous non-vertebral  fracture |  | RR (95% CI): 2.00 (1.90, 2.12) | RR (95% CI): 2.40 (2.15, 2.68) | RR (95% CI): 2.05 (1.97, 2.14) |
|  | Recent use of central nervous  system medication |  | RR (95% CI): 1.94 (1.85, 2.04) | RR (95% CI): 1.66 (1.50, 1.85) | RR (95% CI): 1.33 (1.28, 1.38) |
|  | fall history |  | RR (95% CI): 1.96 (1.79, 2.15) | RR (95% CI): 1.82 (1.47, 2.25) | RR (95% CI): 1.74 (1.60, 1.89) |
|  | Chronic disease/Co-morbidity | (1) Without recent GP visit/ hospitaization  (2) With recent GP visit/ hospitalization | (1) RR (95% CI): 1.39 (1.31, 1.47)  (2) RR (95% CI): 1.88 (1.68, 2.11) | (1) RR (95% CI): 1.63 (1.45, 1.83)  (2) RR (95% CI): 2.38 (1.91, 2.97) | (1) RR (95% CI): 1.18 (1.13, 1.24)  (2) RR (95% CI): 1.29 (1.17, 1.43) |
