# Supplementary material for: Clinical Value of Prognostic Instruments to Identify Patients with an Increased Risk for Osteoporotic Fractures: Systematic Review
Source: PLoS One. 2011 May 18;6(5):e19994. doi: 10.1371/journal.pone.0019994 (PMC3097232; doi:10.1371/journal.pone.0019994)
Supplement: Table S4 — Variables included in the final model. (DOC) [file pone.0019994.s004.doc]

Table 4:Variables included in the final model

| name of variable | number of studies |
| --- | --- |
| age | 26 |
| level of education | 1 |
| history of fracture | 17 |
| Bone mass density (hip…) | 18 |
| bone stiffness ultrasound | 1 |
| maternal (family) history of fractures | 12 |
| past falls | 14 |
| ethnicity, race | 5 |
| heigt loss | 2 |
| BMI (heigt, weight) | 19 |
| smoking | 10 |
| caffeine intake | 1 |
| alcohol | 1 |
| self reported health status | 6 |
| gender | 3 |
| calcium intake | 5 |
| vitamin D level | 1 |
| HRT | 2 |
| age at menarche | 1 |
| early menopause, age at menopause | 4 |
| lactation > 12 months | 1 |
| use of antiepileptic drugs | 3 |
| steroid intake | 4 |
| central nervous medication use | 1 |
| tricyclic antidepressants | 1 |
| use of benzodiazepines | 2 |
| use of hypoglycemic agents | 1 |
| physical activity | 8 |
| chair test | 5 |
| tandem walk | 1 |
| gait speed | 1 |
| locomotion unit | 1 |
| trunk maneuver | 1 |
| quadriceps strength | 2 |
| postural sway | 2 |
| diameter of forearm | 1 |
| ADL | 2 |
| peripheral neuropathy | 1 |
| visual acuity | 1 |
| rheumatoid arthritis | 2 |
| asthma | 1 |
| liver disease | 1 |
| cardiovascular disease | 1 |
| urinary incontinence | 1 |
| diabetes | 2 |
| thyroid disorder (hyperthyr, taking hormones | 3 |
| cognitive status | 3 |
| stroke history | 1 |
| malabsorption | 1 |
| poor circulation | 1 |
| heart rate | 1 |
| back pain | 1 |
| kyphosis | 1 |
