# Supplementary material for: Clinical Value of Prognostic Instruments to Identify Patients with an Increased Risk for Osteoporotic Fractures: Systematic Review
Source: PLoS One. 2011 May 18;6(5):e19994. doi: 10.1371/journal.pone.0019994 (PMC3097232; doi:10.1371/journal.pone.0019994)
Supplement: Table S5 — Search history. (DOC) [file pone.0019994.s005.doc]

Table 5

**Search History**

Ovid MEDLINE(R) In-Process & Other Non-Indexed Citations and Ovid MEDLINE(R)

| |  | **Searches** | **Results** | | --- | --- | --- | | 1 | Validat$.mp. or Predict$.ti. or Rule$.mp. | 350355 | | 2 | (Predict$ and (Outcome$ or Risk$ or Model$)).mp. | 342974 | | 3 | ((History or Variable$ or Criteria or Scor$ or Characteristic$ or Finding$ or Factor$) and (Predict$ or Model$ or Decision$ or Identifi$ or Prognos$)).mp. | 1450528 | | 4 | Decision$.mp. and ((Model$ or Clinical$).mp. or Logistic Models/) | 76293 | | 5 | (Prognostic and (History or Variable$ or Criteria or Scor$ or Characteristic$ or Finding$ or Factor$ or Model$)).mp. | 89262 | | 6 | or/1-5 | 1810733 | | 7 | (bone loss or osteoporos$).ti,ab,hw. | 58419 | | 8 | exp osteoporosis/ | 34303 | | 9 | or/7-8 | 58433 | | 10 | (broken bone$ or fracture$).ti,ab,hw. | 170711 | | 11 | exp Fractures, Bone/ | 113009 | | 12 | or/10-11 | 172410 | | 13 | 6 and 9 and 12 | 4000 | | 14 | limit 13 to yr="1982 - 2009" | 3981 | | 15 | limit 14 to humans | 3522 | |
| --- | --- | --- | --- | --- | --- | --- | --- | --- | --- | --- | --- | --- | --- | --- | --- | --- | --- | --- | --- | --- | --- | --- | --- | --- | --- | --- | --- | --- | --- | --- | --- | --- | --- | --- | --- | --- | --- | --- | --- | --- | --- | --- | --- | --- | --- | --- | --- | --- |
